# The effect of physical activity on sleep disturbance in various populations: a scoping review of randomized clinical trials

**DOI:** 10.1186/s12966-023-01449-7

**Published:** 2023-04-17

**Authors:** Hung-Hsin Huang, Brendon Stubbs, Li-Jung Chen, Po-Wen Ku, Tai-Yi Hsu, Chia-Wei Lin, Yi-Ming Weng, Shih-Hao Wu

**Affiliations:** 1 School of Medicine, College of Medicine, China Medical University, Taichung, Taiwan; 2 Department of Emergency Medicine, China Medical University Hospital, Taichung, Taiwan; 3 Physiotherapy Department, South London and Maudsley NHS Foundation Trust, Denmark Hill, London, SE5 8AZ UK; 4 Department of Psychological Medicine, Institute of Psychiatry, Psychology and Neuroscience, King’s College London, De Crespigny Park, London, SE5 8AF Box UK; 5 Department of Exercise Health Science, National Taiwan University of Sport, 271, Lixing Road, Taichung City, 404 Taiwan; 6 Department of Psychosis Studies, Institute of Psychiatry, Psychology and Neuroscience, King’s College London, 16 De Crespigny Park, London, SE5 8AF UK; 7 Graduate Institute of Sports and Health Management, National Chung Hsing University, 145 Xingda Rd., South Dist, Taichung City, 402 Taiwan; 8 Department of Kinesiology, National Tsing Hua University, Hsinchu, 300 Taiwan; 9 Department of Public Health, China Medical University, Taichung, Taiwan; 10 Doctoral Degree Program in Artificial Intelligence, Asia University, Taichung, Taiwan; 11 Emergency department of Taoyuan General Hospital, MOHW, Taoyuan, Taiwan; 12 Attending Physician of Emergency Department, China Medical University Hospital, Taichung, 404 Taiwan

**Keywords:** Exercise, Insomnia, Cancer, Pregnant women, Sleep environment, Non-pharmacological interventions

## Abstract

**Background:**

Promoting physical activity (PA) in different populations experiencing sleep disturbance may increase population PA levels and improve sleep. This scoping review aimed to examine the effect of various PA intervention strategies on sleep across different populations, identify key sleep outcomes, and analyze knowledge gaps by mapping the relevant literature.

**Methods:**

For this study, we systematically searched articles published till March 2022 from PubMed, Web of Science, Cochrane Library, and Embase databases for randomized clinical trials (RCTs) regarding the effect of physical activity on sleep. Two authors extracted key data and descriptively analyzed the data. Thematic analysis was used to categorize the results into themes by all authors. Arksey and O’Malley’s scoping review framework was used to present the findings.

**Results:**

Twenty-one randomized controlled trials out of 3052 studies were finally included with 3677 participants (2852 females (78%)). Five trials were conducted in healthy working-age adults with sleep disturbance but without the diagnosis of insomnia, five in healthy older adults, two in perinatal women, four in patients with cancer, three in mental illness related subjects, and another two in other disease-related areas. PA interventions were diverse, including walking, resistance training, aerobic exercise, housework, water exercise, basketball, smartphone/tablet "apps", web, online videos or wearable actigraphy, and self-determined exercise. Three major themes were identified: (1) Sleep environment may be important to address prior to instituting PA interventions, (2) All types of PA were effective for improving sleep in all populations studied, (3) Self-tolerated PA is safe for improving sleep in the elderly and in co-morbid or perinatal populations.

**Conclusions:**

PA is effective and safe for improving sleep in both healthy and co-morbid populations with sleep disturbance by increasing daily activity levels using a variety of strategies, even low intensity, such as housekeeping, sit-to-stand repetitions, along with encouraging PA through web pages, videos, and self-goal setting apps. In addition, this scoping review identifies the need for further therapeutic research and future exploration in populations with sleep initiation or sleep maintenance disturbance.

**Supplementary Information:**

The online version contains supplementary material available at 10.1186/s12966-023-01449-7.

## Introduction

Sleep plays an essential role in regulating psychological and physical processes [1]. However, the prevalence of sleeping problems is 56% in the USA, 31% in Western Europe, and 23% in Japan [2]. Poor sleep can lead to daytime sleepiness and dysfunction [3], with sleep-related accidents a common cause of death and injury worldwide [4]. Moreover, sleep disturbances can increase the risks of depression [5], dementia [6], diabetes [7], cardiovascular disease [8], and mortality [9].

Treatment options for sleep disturbances range from lifestyle modification to medication. Non-pharmacological therapy is increasingly used to help address sleep disturbance, including massage [10], aromatherapy [11], cognitive-behavioral therapy [12], sleep hygiene, and physical activity (PA) [13, 14]. PA is beneficial for mental [15] and physical health [16], including sleep improvement [17] and reduced mortality [18].

A recent umbrella review examined whether PA enhances sleep outcomes across the lifespan as well as among individuals with sleep disorders [19]. It found strong evidence that both acute bouts of PA and regular PA improved sleep outcomes [19]. Proposed mechanisms include changes in body temperature, heart rate, mood, and the results of increased energy expenditure, along with secretions of brain-derived neurotropic factor, growth hormone [20, 21], serotonin [22, 23], and melatonin [24].

Though it is well-documented that exercise is beneficial to health and cognition, only about 25% of US adults meet the recommended weekly PA guidelines (150 min of moderate-intensity aerobic activity and/or 2 days of muscle-strengthening) [25]. Middle-aged and older adults are less likely to meet these recommendations than younger adults [25]. Light‐intensity or daily PA, such as walking or housekeeping, is a low-impact activity that is easy to practice, safe for all age groups, and easy to encourage throughout the day.

Many studies have demonstrated the effectiveness of various intensities and types of exercise in improving sleep both in healthy populations [26–29], and some other specific groups, such as people with fibromyalgia [30], and children and youth [31]. However, due to the volume of studies on PA and sleep, some evidence of confusion and conflict exists [32–35]. Most of these reviews are extracted from cross-sectional studies and can thus only comment on associations. To our knowledge, few reviews have focused on the effects of PA, which is easily implemented in daily life, on sleep across different populations.

Our study aimed to evaluate the effect of various PA intervention strategies on sleep across different populations published with randomized controlled trials (RCTs), identify sleep outcomes using Buysse's sleep health framework [36], and analyze knowledge gaps by mapping the relevant literature.

## Materials and methods

Based on the study aim, a scoping review was determined to be the most appropriate method, since it was too broad to address via a traditional systematic review/meta-analysis. A scoping review is an appropriate methodology for reviewing large bodies of literature to generate an overview of a research topic [37]. This study used the five-stage methodological framework for scoping studies developed by Arksey and O’Malley [38], including: (1) identifying the research question; (2) identifying relevant studies; (3) selecting studies; (4) charting the data; and (5) collating, summarizing, and reporting the results.

### Identifying the research questions

The research question was purposefully refined to encompass the extensive range and nature of existing research activities in the literature as follows: What is the currently available evidence related to PA for sleep disturbance in various populations? The following research questions guided this review: (1) What are the characteristics of the subjects? (2) What are the strategies for PA? (3) What are the programs for PA intervention? (4) What are the outcome measurements and the results? All of the above questions prompted the final key question: Can PA really relieve sleep disturbance?

### Identifying relevant studies

Relevant published RCTs involving the effect of PA and sleep were identified on 30^th^ March 2022, using the following online databases: PubMed, Web of Science, Cochrane Library, and Embase. To include all possible sleep outcomes and any unexpected light‐intensity PA interventions, the following keywords were searched in the titles: “physical activity” AND “sleep.” We did not use other terms, such as walking or housekeeping, to avoid limiting the scope of searches for PA. In addition, “randomized controlled trial” filter was applied to narrow our search. There was no restriction on the original language of the study, and an automatic page translation to English was applied. All the retrieved studies were manually screened by the authors Huang and Wu for relevance. The studies were saved in reference manager software (EndNote 20, Thomson Reutersc, New York, NY, USA).

### Selecting studies

Relevant studies published till 30^th^ March 2022 were identified during the search. After removing duplicates, articles were screened by blinding the results only to show the title and abstract, and then screened using the Endnote referencing software. Articles were included if (i) the included participants were those with sleep disturbance ; (ii) the intervention was related to PA; (iii) the outcome was sleep; and (iv) the research design was an RCT. Articles were excluded if they (i) were observational studies; (ii) had no control group, or if they (iii) studied the effects of other medications or supplements, (iv) the outcome was not sleep, (v) were gray literature, such as seminar posters or abstracts only, (vi)involved animal studies, or (vii) were protocols.

For articles that met initial eligibility criteria, the full article was retrieved and assessed by two independent reviewers to ensure consistent application of the eligibility criteria for inclusion. Disagreements about the study eligibility of the sampled articles were discussed between the two reviewers until a consensus was reached. Because of the present lack of guidelines on the reporting of scoping reviews, the Preferred Reporting Items for Systematic Reviews and Meta-Analysis (PRISMA) guidelines [39] was used to report the flow of the articles included in this review.

### Charting the data

Key items of information from the included articles were charted onto a form developed based on the research questions. We used Cohen’s d as an indicator of effect size and interpreted effects as small (0.2), medium (0.5) or large (0.8) [40]. If the study did not report effect sizes directly, we attempted to calculate effect sizes from the available data. If the available data could not be obtained for the calculation of effect size, we tried to find the effect size of the particular study reported from meta-analyses in the literature. If it still could not be found, it was recorded as not reported. Two independent reviewers (Huang and Wu) extracted information from each article across each data extraction category. The reviewers met to compare the extracted information and discrepancies were discussed between the two reviewers until a consensus was reached.

### Assessment of the quality of the methodologies of the studies

Quality Assessment of Controlled Intervention Studies (QACIS) was used to evaluate the methodological quality of the included studies [41]. For the methodological quality assessment, the items were rated as 1 (meets the criteria), 0 (does not meet the criteria), or N/A (not applicable). The final score was calculated based on the total sum of the scored items divided by the total number of items scored, expressed as a percentage. Two authors (Huang and Wu) independently assessed the quality of the studies, with any disagreements discussed until a consensus was reached (as Additional file 1, & 2); before this, the Cohen’s kappa score was 0.71, indicating a substantial level of interrater reliability. The NHLBI tool does not have any set threshold for quality scores. Nonetheless, using general study quality assessment guidelines, the studies were characterized as having either low (≤50%), good (51–75%), or excellent (>75%) methodological quality [42, 43].

### Collating, summarizing, and reporting the results

After extraction, separation, grouping, and abstraction of the text findings, two reviewers independently categorized the findings into different groups. To address the multiple factors related to sleep and PA in different populations, the current review uses the Buysse's framework of sleep health [36] to recognize measurable characteristics of sleep in different populations with diverse PA interventions. The framework uses five dimensions of sleep (SATED): Satisfaction, Alertness (during waking hours), Timing, Efficiency, and Duration [36].

Upon further discussion, prominent themes constituting frequently reported overlapping data and key factors resulting in negative findings were selected from the authors’ independent analyses, renamed, and included in the final analysis. To enhance reflexivity, the first phase of analysis was carried out independently by the two authors. Any discrepancies between the two authors over the thematic analysis were clarified by consulting the wider research team in the meeting until consensus on the final results was reached. The authors of this review represented different backgrounds, to include varied perspectives when summarizing the available evidence on the application of PA for sleep.

## Results

### Main results

**Fig. 1:**
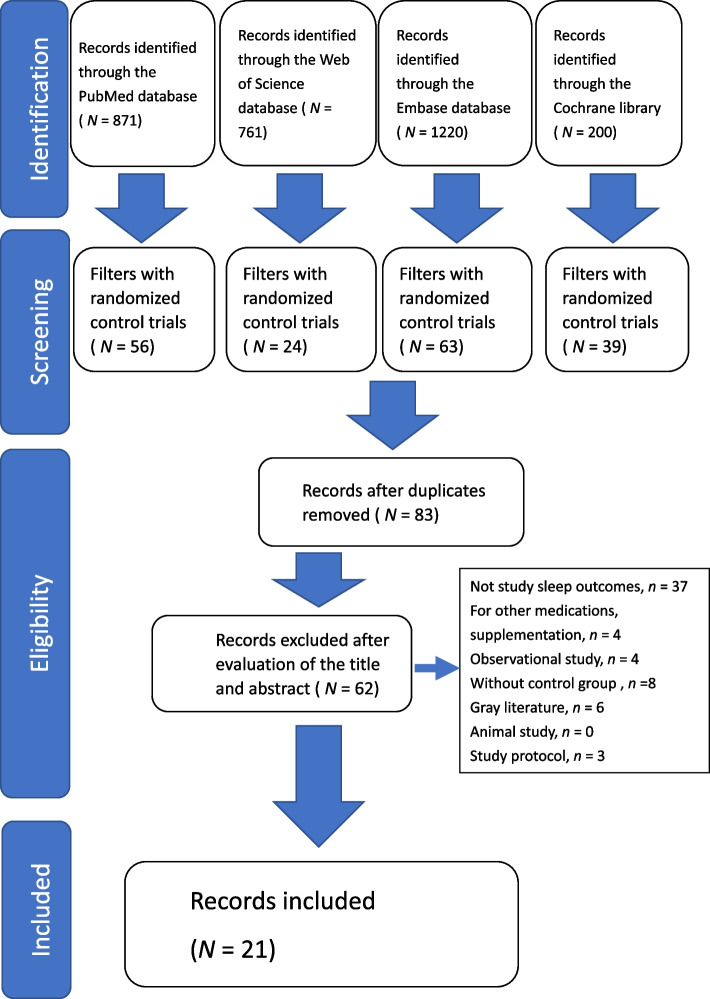
Study selection flowchart. According to the Preferred Reporting Items for PRISMA guidelines

A study selection flowchart is presented in Fig. 1, according to the Preferred Reporting Items for PRISMA guidelines [39]. In total, 3052 publications were identified. Of these, 2870 were excluded, leaving 83 publications for potential inclusion. Sixty-two of these studies were excluded because they did not study sleep outcomes (*n* = 37), studied other medications or supplementations (*n* =4), were observational study (*n* = 4), had no control group (*n* = 8), were gray literature (*n* = 6) or just a study protocol (*n* = 3). Finally, 21 studies were identified as eligible for inclusion.

### Included studies

All of the 21 trials included were conducted between 1995 and 2021. In total there were 3677 participants (2852 female (78%)). The sample size of studies ranged from 10 to 1635 participants. Seven trials included only female subjects [44–50], while the remaining 14 included both genders, although in 13 of these 14 trials most participants were female (72%). One study mainly including males was conducted in children with an autism spectrum disorder [51]. The studies were categorized into six groups according to the different subjects. The key information and findings extracted from these studies were summarized by distinct groups in Tables 1, 2, 3, 4, 5 and 6.

**Table 1:** Five RCTs investigated the effect of PA on sleep in healthy working-aged adults

Authors (year)	Design	Participants	Country	Intervention duration	Intervention and measures	Outcome measures (Sleep related)	Main findings (sleep-related)
Bisson et al. (2019) [52]	RP	59 healthy adults 72%female 49.43(± 8.40) years old	USA	4 weeks	Increasing participants’ daily steps,2000 steps Actigraphy with Fitbit Zip	Self-reports of sleep quality and duration PSQI	Daily active minutes were positively related to sleep quality (*p* < 0.05), but not duration, in women than men More active than average, they reported better sleep quality and duration in both genders
Murawski et al. (2019) [53]	RP	160 adults, 80% female 18 − 55 years old reported being insufficiently physically active (< 90 min/week), and poor sleep quality	Australia	12 weeks	The Balanced app, which provided a platform for personalized goal setting, daily logging with dynamic feedback, Active Australia Questionnaire	PSQI, Sleep Hygiene Index, Sleep Timing Questionnaire, Insomnia Severity Index, Epworth Sleepi- ness Scale	The intervention group reported better overall sleep quality (*P =* 0.009), subjective sleep quality (*P =* 0.017), sleep onset latency (*P =* 0.013), waketime variability (*P =* 0.018), sleep hygiene (*P =* 0.027), insomnia severity (*P =* 0.002)
McDonough et al. (2021) [54]	RP	64 young adults 75% female 22.8 ± 3.4 years old	America	12 weeks	Weekly aerobic and muscle-strengthening physical activity videos, ActiGraph accelerometers	ActiGraph accelerometers	YouTube-delivered PA intervention may help foster clinically meaningful improvements in young adults’ free-living moderate-to-vigorous PA, muscle-strengthening PA frequency, sleep efficiency, (F(1, 62) = 10.75–77.67, *p* < 0.001–0.002,η_p_^2^ = 0.15–0.56)
Rayward et al. (2020) [55]	RP	275 middle-aged adults 82.9% female 40–65 years old who report physical inactivity and poor sleep quality	Australia	3 months	Smartphone/tablet “app” to aid goal setting and self-monitoring physical activity and/or sleep hygiene behaviors, Pedometer	PSQI	No differences in sleep quality between Physical Activity and Sleep Health group and Sleep Health Only group
Chee et al. (2019) [44]	RP	26 Asian American (Chinese or Korean) midlife women aged 40 to 60 years	America	3 months	Web-based physical activity promotion programs( WPAPP), The Physical Activity Assessment Inventory (PAAI)	The Sleep Index for Midlife Women (SIMW)	The WPAPP is potentially beneficial in decreasing Asian American midlife women’s sleep-related symptoms The intervention group showed a trend of decrease in the total numbers of psychological (*P =* 0.0956) and total sleep-related symptoms (*P =* 0.0733)

*RP* Randomized parallel, *RC* Randomized crossover, *PSQI* Pittsburgh sleep quality index, *PA* Physical activity, *WPAPP* Web-based physical activity promotion programs, *PAAI* The physical activity assessment inventory, *SIMW* The sleep index for midlife women

**Table 2:** Five RCTs investigated PA in healthy older adults

Authors (year)	Design	Participants	Country	Duration	Intervention and measures	Outcome measures (Sleep related)	Main findings (Sleep-related)
Alessi et al (1995) [56]	RP	65 nursing home residents 85% female Mean age: 84.8 years old urinary incontinence or were physically restrained	USA	9 weeks	Sit-to-stand repetitions, walking, wheelchair propulsion, rowing machine Mobility endurance (maximum time walking or wheeling) and motion sensors (Caltrac)	Nighttime sleep: wrist activity monitors Daytime sleep: timed behavioral observations of sleep versus wakefulness performed every 15 min during the day	No improvements in sleep in the intervention versus control groups Even among a subgroup of intervention subjects who showed a 30% or greater improvement in mobility endurance, sleep did not improve at follow-up compared with baseline
Alessi et al (1999) [57]	RP	29 incontinent nursing home residents 90% female mean age:88.3 years old	USA	14 weeks	Functional incidental Training, involved a structured series of arm and leg exercises, sit-to-stands, and walking or wheelchair propulsion Daytime physical activity monitors and structured physical function assessments	nighttime wrist activity monitors to estimate nighttime sleep; and timed day- time behavioral observations of sleep versus wakefulness	Nighttime percent sleep (time asleep over time monitored in bed at night) increased in intervention subjects from 51.7% at baseline to 62.5% at follow-up compared with 67.0% at baseline to 66.3% at follow-up in controls (MANOVA, group by time, *F* = 4.42, *P =* .045, *df* = 27)
Seol et al. (2021) [45]	RC	10 healthy older women 65–79 years old	Japan	30 min	Low-intensity physical activity for 30 min, either aerobic exercise (70 steps per minute), housework (at the same intensity), ActiGraph GT3X-BT	Oguri-Shirakawa-Azumi sleep inventory, Middle-Aged and Aged version (OSA-MA) questionnaire polysomnography	A significant difference in sleep latency (14.2 ± 19.1, 9.9 ± 15.6, and 4.2 ± 3.5 min for control, housework, and aerobic exercise, respectively; ANOVA *P =* 0.011) among the trials The total score on OSA-MA was significantly higher after aerobic exercise (91.0 ± 5.4, 88.1 ± 6.9, and 108.6 ± 5.9 points for control, housework, and exercise, respectively)
Bademlim et al. (2019) [58]	RP	60 elderly individuals with mild cognitive impairment, 58% female over 65 years old	Turkey	20 weeks	Physical Activity Program (10‐minute of warm‐up activities,20‐minute of rhythmic exercises, 10‐minute of cool down exercises and 40‐minute of free walking), metabolic equivalent level	PSQI	Sleep quality of elderly individuals improved thanks to a 20‐week program of regular physical activity
Vazfragoso et al. (2015) [59]	RP	1635 older adults who were sedentary 67% female 70 to 89 years old	USA	30 months	walking with a goal of 150 min/week and strength, flexibility, and balance training, accelerometry	Insomnia Severity Index (ISI), Epworth Sleepiness Scale, PSQI	Structured physical activity resulted in a lower likelihood of developing poor sleep quality (PSQI > 5) over the intervention period than health education but had no effect on prevalent cases of poor sleep quality or on sleep–wake behaviors evaluated using the ISI or ESS

*RP* Randomized parallel, *RC* Randomized crossover, *PSQI* Pittsburgh sleep quality index, *ISI* Insomnia severity index, *OSA-MA* Oguri-shirakawa-azumi sleep inventory, middle-aged and aged version

**Table 3:** Two RCTs investigated PA in sleep in pregnancy and the post-partum women

Authors (year)	Design	Participants	Country	Duration	Intervention and measures	Outcome measures (Sleep related)	Main findings (sleep-related)
Rodriguez-Blanque et al (2018) [46]	RP	140 pregnant women aged 21–43 years	Spain	week 20 to week 37(18 weeks)	Water Exercise in Pregnancy program	PSQI	Water Exercise in Pregnancy method improves the quality of sleep in pregnant women, both subjectively and in terms of latency, duration and efficiency
Hawkins et al. (2019) [47]	RP	204 Hispanic postpartum women at risk for type 2 diabetes, mean age of 27.8	USA,	46 weeks postpartum	Lifestyle intervention Pregnancy Physical Activity Questionnaire	PSQI	sports/exercise was associated with lower odds of very poor sleep quality (PSQI > 10) (OR = 0.18, 95% CI¼ 0.05 to 0.69). Sports/exercise (OR = 0.05, 95% CI¼ 0.01 to 0.26) and vigorous intensity PA (OR = 0.13, 95% CI¼ 0.04 to 0.42) were associated with lower odds of short (vs normal) sleep duration No statistically significant associations between any measure of physical activity and sleep quality as a continuous outcome

*RP* Randomized parallel, *PSQI* Pittsburgh sleep quality index, *PA* Physical activity, *OR* Odds ratio

**Table 4:** Four RCTs investigated the effect of PA on sleep in people with cancer

Authors (year)	Design	Participants	Country	Duration	Intervention and measures	Outcome measures (Sleep related)	Main findings (sleep-related)
Roveda et al (2017) [48]	RP	40 breast cancer women 55.2 (± 6.8) years for the intervention group and 58.2 (± 6.4) years for the control group	Italy	3 months	2 sessions of 1-h brisk walking per week for 3 months The heart rate monitor	Actigraph Actiwatch	Control group showed a generalized deterioration of their sleep behavior, whereas the intervention group maintained their sleep behavior more stable
Rogers et al. (2017) [49]	RP	222 post-primary treatment breast cancer survivors (mean age of 54.4 years)	USA	3 months	physical activity behavior change intervention	PSQI and wrist-worn accelerometer	intervention group significantly improved PSQI global sleep quality when compared with usual care at 3 months( *p* < .001) and 6 months( *P =* .01)
Rastogi et al. (2020) [60]	RP	50 breast and colorectal cancer survivors 96% female aged 54.4 ± 11.2 years	USA	12 weeks	12-week multi-component physical activity module Gradually increase their MVPA to 150 min/week and daily steps to 10 000 Fitbit tracker ActiGraph GT3X +	Patient Reported Outcomes Measurement Information System's (PROMIS) sleep measures, 8-item short forms	Greater reduction in both sleep disturbance and impairment among the cancer survivors in the intervention group from baseline to 12-weeks relative to the comparison group Both scales were associated with medium effect sizes, with impairment (d = − 0.62) improving more than disturbance (d = − 0.46)
Nguyen et al. (2021) [50]	RP	83 inactive, postmenopausal breast cancer survivors(aged 62 ± 6.4 years)	Australia	12 weeks	a wearable-based physical activity intervention (Garmin Vivofit2® coupled with behavioral feedback, goal setting, and health coaching)	Wrist-worn actigraphy and PSQI	After intervention, primary intervention participants had greater improvements in WASO (− 5.7 min, 95% CI − 11.7 to − 0.2) and NWAKE compared with the waitlist arm (− 2.0, 95% CI − 3.6 to − 0.4) At 24 weeks later, within-group improvements were observed for SE (both groups), WASO (both groups), NWAKE (primary intervention group only), total PSQI score (primary intervention group), and sleep efficacy (primary intervention group)

*RP* Randomized parallel, *RC* Randomized crossover, *PSQI* Pittsburgh sleep quality index, *MVPA* Moderate vigorous physical activity, *WASO* Wake after sleep onset, *NWAKE* Number of awakenings, *PROMIS* Patient reported outcomes measurement information system's

**Table 5:** Three RCTs investigated PA in sleep in subjects with mental illness

Authors (year)	Design	Participants	Country	Duration	Intervention and measures	Outcome measures (Sleep related)	Main findings (Sleep-related)
Hartescu et al (2015) [61]	RP	41 patients, 73% female 59.80 ± 9.46 years old inactive people with insomnia	UK	6 months	moderate-intensity physical activity as ‘brisk walking’ totaling at least 150 min per week, NewLife NL-1000 activity monitor	Insomnia Severity Index	physical activity group showed significantly reduced insomnia symptom severity (*P =* 0.03), with an average reduction of four points on the Insomnia Severity Index
Wang et al. (2015) [62]	RP	71patients with chronic insomnia 64.8% female 22–58 years old	China,	4 weeks	Physical activity counseling combined with sleep restriction (PASR), International Physical Activity Questionnaire (IPAQ)	Insomnia severity index, sleep diary	PA counseling based on 5A model (assess, advise, agree, assist, and arrange) combined with SR cannot only effectively increase the PA levels but also improve the sleep quality for patients with chronic insomnia. (all *p* < 0.05)
Tse et al. (2019) [51]	RP	40 children with autism spectrum disorders 20% female 8–12 years old	China	12 weeks	12-week basketball skill learning	actigraphy accelerometer	a significant improvement in sleep efficiency, sleep onset latency, and sleep duration in the intervention group but not in the control group during weekdays

*RP* Randomized parallel, *PASR* Physical activity counseling combined with sleep restriction, *IPAQ* International physical activity questionnaire, *PA* Physical activity

**Table 6:** Two RCTs investigated PA in sleep in subjects with other health conditions

Authors (year)	Design	Participants	Country	Duration	Intervention and measures	Outcome measures (Sleep related)	Main findings (sleep-related)
Freburger et al. (2010) [63]	RP	346, older adults with arthritis 87.5% female Mean age: 69.74 years old	USA,	8 weeks	low-to-moderate-intensity physical activity, People With Arthritis Can Exercise (PACE) program	self-rated sleep quality	At 8 weeks, the intervention group reported fewer days waking up tired and fewer days waking up at night and/or having poor mental health Treatment effects were not maintained at 3 and 6 months
Cho et al. (2018) [64]	RP	57 maintenance hemodialysis patients 54.4% female mean age 57 years old	South Korea,	12 weeks	A stationary bike was used for aerobic exercise (AE) and a TheraBand ®/theraball for resistance exercise (RE) triaxial accelerometer	triaxial accelerometer	Average movement index (%) decreased significantly in the AE (*P =* 0.006), RE (*P =* 0.001), and CE groups (*P =* 0.02) at 12 weeks compared with baseline The average sleep fragmentation index (%), indicating poor Sleep quality, decreased significantly at 12 weeks compared with baseline in the AE (*P =* 0.03) and RE groups (*P =* 0.01)

*RP* Randomized parallel, *RC* Randomized crossover, *PSQI* Pittsburgh sleep quality index, *AE* Aerobic exercise, *RE* Resistance exercise

### Methodological quality appraisal

The methodological quality of these trials was excellent (66.7% [14/21]), good (28.6% [6/21]), and low (4.8% [1/21]; Table 1S). The average score of the QACIS was 77.89%, indicating excellent methodological quality. Studies were downgraded for reasons such as insufficient control conditions and/or small sample size.

### Geographic location of included studies

Research studies were identified from nine countries, with most (ten studies [44, 47, 49, 52, 54, 56, 57, 59, 60, 63]) from the USA. The other studies were conducted in Australia (*N*=3) [50, 53, 55] , Italy (*N*=1) [48] , Spain (*N*=1) [46], South Korea (*N*=1) [64], Japan (*N*=1) [45], Turkey (*N*=1) [58], UK (*N*=1) [61] and China (*N*=2) [51, 62].

### Physical activity intervention

The types of PA interventions were diverse. They included studies on walking (*N*=10) [48, 49, 52, 56–62], resistance training (*N*=3) [53, 54, 64], aerobic exercise (*N*=3) [45, 54, 64], housework (*N*=1) [45], water exercise (*N*=1) [46], basketball (*N*=1) [51], smartphone/tablet "apps", web, online videos, or wearable actigraphy with feedback and goal setting (*N*=5) [44, 50, 53–55], other unspecified mixed exercise program (*N*=2) [47, 63], and self-determined exercise (*N*=4) [44, 47, 50, 55]. Of the PA interventions, eleven trials (52%) were well structured with defined frequency, intensity, time, type, and duration and were rigorously monitored for PA interventions (four in older adults, one in pregnant women, one in people with arthritis, one in people with hemodialysis, one in people with cancer, three in people with psychiatric disorders) [45, 46, 48, 51, 56–58, 61–64], all of which were exercise interventions rather than wider PA. However, these exercises were still easy to implement in daily life. The supervision of PA was all for the safety of the subjects.

The intensity of the PA interventions was also diverse. The included studies utilized low/light intensity PA mainly in older populations (*N*=5) [45, 52, 56–58], light-to-moderate PA (*N*=2, one in subjects with arthritis, and another in older adults) [59, 63], moderate PA (*N*=5, two in perinatal women, two in subjects with insomnia, one in subjects with breast cancer) [46–48, 61, 62], moderate-to-vigorous-intensity PA(*N*=6, three in working age adults, one in subjects receiving hemodialysis, two in subjects with cancer) [49, 53–55, 60, 64], and unspecified or not reported (*N*=2) [44, 51].

Regarding the frequency of interventions, the studies included daily self-monitoring (app, telephone, web) (*N*=3) [52, 53, 55], daily practice with a coach (*N*=2) [56, 57], practice with a coach twice a week (*N*=3) [48, 51, 63], three sessions a week (*N*=3) [46, 49, 64], at least five times per week (*N*=3) [59, 61, 62], irregular (4 days a week, 3 days a week, 7 days a week, in accordance with ergonomics and the physiological characteristics of individuals older than 65 years) (*N*=1) [58], and subjects self-determined under goal-directed targets (*N*=5) [44, 47, 50, 54, 60].

The duration of set interventions ranged from 5 to 80 minutes. These included 9.5% (*N*=2) lasting less than 30 minutes [56, 57], 14.3% (*N*=3) 30 minutes [45, 61, 62], 4.8% (*N*=1) 40 minutes [64], 4.8% (*N*=1) 45 minutes [51], 4.8% (*N*=1) 50 minutes [59], 14.3% (*N*=3) one hour [46, 48, 63], 4.8% (*N*=1) 80 minutes [58], while the rest (42.9% (*N*=9)) carried out goal-directed interventions with the timing being at their own discretion [44, 47, 49, 50, 52–55, 60].

Characteristics of PA interventions are presented in Tables 1, 2, 3, 4, 5 and 6 and Additional file 3. There was only one “acute” PA intervention which entailed low-intensity PA “once” for 30 minutes, either aerobic exercise (70 steps per minute, utilizing a step-up platform), housework (at the same intensity), or remaining sedentary for 3 hours before bedtime (control condition) [45]. The other studies were “chronic” interventions, length of duration of interventions ranged from 4 weeks to 30 months. There were 67% (*N*=14) between 4 to 12 weeks [44, 48–56, 60, 62–64], 19% (*N*=4) between 14 to 24 weeks [46, 57, 58, 61], and 10% (*N*=2) above 6 months.

### Sleep outcome measures

As shown in Table 7, Sleep outcomes of the included studies were measured using varied methods. Six studies (29%) used objective measurements only, such as actigraphy accelerometer or polysomnography [48, 51, 54, 56, 57, 64], while 12 studies (57%) used subjective measurements only [44, 46, 47, 52, 53, 55, 58–63], including the Pittsburgh Sleep Quality Index (PSQI) (*N*=9) [46, 47, 49, 50, 52, 53, 55, 58, 59], the Insomnia Severity Index(*N*=4) [53, 59, 61, 62], the Sleep Index for Midlife Women (*N*=1) [44], the Patient Reported Outcomes Measurement Information System (*N*=1) [60], and the Oguri-Shirakawa-Azumi sleep inventory [45]. These subjective sleep measurements were multi-dimensional. The remaining three studies (14%) employed both subjective and objective measurements [45, 49, 50].

**Table 7:** The effect of physical activity on sleep

Authors (year), subjective(S) /objective(O)	Subjects/ (QACIS score)	sleep quality	Total sleep time /actual sleep time	Sleep efficiency	Sleep onset latency	Im-mobility time/mobility index	Wake after sleep onset	sleep fragmentation index/ waketime variability	Sleepiness in the day time/day time dysfunction	PSQI	ISI	PROMIS/ SIMW/ OSA-MA
Sleep dimensions		Satisfaction	Duration	Efficiency	Efficiency	Efficiency	Efficiency	Efficiency	Alertness	Alertness, Efficiency, Satisfaction	Alertness,Efficiency, Satisfaction	
Bisson et al. (2019) (S) [52]	Healthy middle age adults/ (79)	**S**	**N**							**S**		
Murawski et al. (2019) (S) [53]	Healthy middle age adults/ (71)	**M**			**NR**			**M**		**M**	**M**	
McDonough et al. (2021) (O) [54]	Healthy Young adults/ (93)		**N**	**M**								
Rayward et al. (2020) (S) [55]	Healthy middle age adults/ (86)	**N**	**N**	**N**	**N**				N	**N**		
Chee et al. (2019) (S) [44]	Asian American midlife women/ (79)											**N( SIMW)**
Alessi et al (1995) (O) [56]	impaired nursing home residents/(86)		**N**					**N**	**N**			
Alessi et al (1999) [57] (O)	Incontinent nursing home residents/(64)			**L**					NR			
Seol et al. (2021) (S/O) [45]	healthy older women/ (86)		N	**N**	**L:exercise** **S:housework**		**N**					**L:exercise (OSA-MA)**
Bademlim et al. (2019) (S) [58]	elderly individuals with mild cognitive impairment/(86)	**L**								**L**		
Vazfragoso et al. (2015) (S) [59]	people aged 70 to 89 who were sedentary/(93)								**N**	**S**	**N**	
Rodriguez-Blanque et al.(2018)(S) [46]	pregnant women/ (79)	**L**	**L**	**L**	**L**					**L**		
Hawkins et al. (2019) (S) [47]	Hispanic postpartum women/ (57)	**L**	**M**							**L**		
Freburger et al. (2010) (S) [63]	older adults with arthritis/ (71)						**S**		**M**			
Cho et al. (2018) (O) [64]	maintenance hemodialysis patients/ (43)		**N**	**N**		**L**	**N**	**L**				
Nguyen et al. (2021) (S/O) [50]	breast cancer/ (71)			**M**			**M**			**M**		
Rastogi et al. (2020) (S) [60]	breast and colorectal cancer/ (79)											**M( PROMIS)**
Rogers et al. (2017) (S/O) [49]	breast cancer/ (86)	**M**		**N**	**N**					**S/M**		
Roveda et al. (2017) (O) [48]	breast cancer/ (71)		**S**	**L**	**M/L**	**L**	**M**	**M**				
Hartescu et al. (2015) (S) [61]	insomnia/(86)										**L**	
Wang et al. (2015) (S) [62]	insomnia/(86)		**N**	**M**	**N**		**N**				**N**	
Tse et al. (2019) (O) [51]	children with autism spectrum disorders/ (86)		**S**	**L**	**M**		**L**					

*ISI* Insomnia severity index, *PSQI* Pittsburgh sleep quality index, *PROMIS* Patient reported outcomes measurement information system, *SIMW* The sleep index for midlife women, *OSA-MA* Oguri-shirakawa-azumi sleep inventory, Middle-Aged and Aged version, *ESS* Epworth sleepiness scale, N No effect, *NR* Positive effect, but Not reported effect size, *S* Small effect size(Cohens’ d:close to 0.2), *M* Medium effect size(Cohens’ d:close to 0.5), *L* Large effect size(Cohens’ d:close to 0.8)

The most evaluated sleep dimension was efficiency, such as sleep efficiency (*N*=11), while the least used was the immobility time/activity index (*N*=2), which is a sub-measure of sleep efficiency. The greatest effect size was observed in changes in PSQI-measured sleep quality (Large effect size, *N*=3; Medium effect size, *N*=2) and sleep efficiency (Large effect size, *N*=2; Medium effect size, *N*=3), while the smallest effect was observed on duration (total sleep time) (No effect, *N*=7; Small effect size, *N*=2). Therefore, PA trials are more effective in improving efficiency than duration of sleep. There was a lack of trials examining alertness and sleep timing.

### Participant characteristics

The studies were categorized into six groups based on their participants. Five studies looked at healthy working age adults, ranging in age from 18 to 65, with an average age of 45.4. Four studies had a majority female sample [52–55], and another included only females [44]. Five studies examined older adults, with an average age of 77.4 years [45, 56–59]. Two studies were related to the perinatal period, both during pregnancy [46] and postpartum [47], with a mean age of 29.2 years. Four focused on cancer, three with women with breast cancer [48–50] and the other with women with breast and colorectal cancers [60], ranging in age from 18 to 75, and the mean age of the four studies was 56.24 years old. All participants of these four studies completed the primary treatment (surgery, chemotherapy, radiation therapy), ongoing hormone therapy acceptable, but not scheduled for and not currently undergoing chemotherapy. Three studies investigated psychiatric patients , two with chronic insomnia but no history of any other major psychiatric disorder [61, 62] while another study included pediatric participants aged 8–12 years with a diagnosis of autism spectrum disorder [51]. Other studies investigated arthritis [63] and patients undergoing hemodialysis [64].

### Study outcomes

#### Healthy working age adults

Five RCTs investigated the effect of PA on sleep in healthy working-aged adults, (Table 1, at the end of the text ). Generally, the studies showed that PA was positively related to sleep quality. Bisson et al. proved that on days when participants accumulated more daily steps than average, they reported significantly better sleep quality and longer sleep duration, while averaged across the month, daily active minutes were positively related to sleep quality, but not duration [52]. Murawski et al. revealed that subjects in an intervention group using the Balanced App which provided a platform for personalized goal setting and daily logging with dynamic feedback for 12 weeks, reported improved overall sleep quality (p=0.009), subjective sleep quality (p=0.017), sleep onset latency (p=0.013), waketime variability (p=0.018), sleep hygiene (p=0.027) and insomnia severity (p=0.002) [53]. However, Rayward et al. found that an adjunctive App-based PA intervention did not improve sleep quality [55]. Chee et al. reported that Web-based PA promotion programs were also potentially beneficial in decreasing Asian American midlife women’s sleep-related symptoms (*p*=0.0733) [44]. Besides, McDonough et al. found that YouTube-delivered PA intervention may help foster clinically meaningful improvements in working age adults’ free-living moderate-to-vigorous PA, muscle-strengthening PA and sleep efficiency (F(1, 62) = 10.75-77.67, *p* < 0.001-0.002,η_p_^2^ = 0.15-0.56) [54]. (Table 1, at the end of the text).

#### Healthy older adults

Five RCTs investigated PA in healthy older adults, (Table 2, at the end of the text). Alessi et al. (1995) found that increasing daytime PA alone did not improve sleep in nursing home residents [56]. In a further study in 1999 the same team found that combining increased PA with an improved night-time nursing home environment resulted in greater improvement in sleep efficiency compared with the nighttime program alone [57]. Likewise, Bademlim et al. revealed that sleep quality of elderly nursing home residents improved thanks to regular PA [58]. Vaz Fragoso et al.’s study of 1635 people aged from 70 to 89 years old found that structured PA resulted in a lower likelihood of developing poor sleep quality but had no effect on existing poor sleep quality or on sleep-wake behaviors, as evaluated using the Insomnia Severity Index or the Epworth Sleepiness Scale [59]. Seol et al. found that low-intensity exercise in the evening induced a positive effect on objective sleep latency and self-reported sleep quality. Although the same intensity of housework results in a similar physiological response, housework resulted in lower self-reported sleep quality than exercise [45] (Table 2).

### PA in pregnancy and the post-partum period

Two RCTs investigated PA in sleep in perinatal women, as shown in Table 3, at the end of the text. Rodriguez-Blanque et al. revealed that water exercise through gestational weeks 20 to 37 was beneficial for sleep in pregnant women between the ages of 21 and 43, with the difference being more pronounced in women who were overweight or obese [46]. Hawkins et al. found that participating in sports or exercise was associated with lower odds of very poor sleep quality and short sleep duration in postpartum women, supporting the hypothesis that PA has a beneficial relationship with sleep in the postpartum period [47, 65] (Table 3, at the end of the text).

### Subjects with cancer

Four RCTs investigated the effect of PA on sleep in people with cancer, as shown in Table 4, at the end of the text. Roveda et al. evaluated sleep quality using objective sleep measures including actigraphy/ accelerometer in breast cancer patients. They found aerobic PA had a protective effect against factors promoting sleep disruption, with the potential to render sleep behavior less sensitive to detrimental environmental factors [48]. In contrast, Rogers et al. revealed that while a PA behavior change intervention significantly improved subjective PSQI global sleep quality in post-primary treatment breast cancer survivors, this was not reflected in objective measurement with accelerometers [49, 66, 67]. Nguyen et al. proved that a wearable-based PA intervention (Garmin Vivofit2®) coupled with behavioral feedback, goal setting and health coaching achieved greater improvements in WASO (wake after sleep onset) and NWAKE (number of awakenings) compared with a waitlist arm [50, 68]. Rastogi et al. found that a 12-week multi-component PA module resulted in greater reduction in sleep disturbance and impairment among breast and colorectal cancer survivors. In addition, that intervention had a moderate effect on several psychosocial and quality of life outcomes of survivors [60] (Table 4, at the end of the text).

### Subjects with mental illness

Three RCTs investigated PA in sleep in subjects with mental illness, as shown in Table 5, at the end of the text. Hartescu et al. found that PA significantly reduced the severity of insomnia symptoms with an average reduction of four points on the Insomnia Severity Index [61]. Wang et al. demonstrated that PA counseling based on the 5A model (assess, advise, agree, assist, and arrange) combined with sleep restriction therapy could not only effectively increase PA levels but also improve sleep efficiency for patients with chronic insomnia, when compared to sleep restriction therapy alone [62] (Table 5, at the end of the text) Tse et al. conducted a study of children with autism spectrum disorders and showed a significant improvement in sleep efficiency, sleep onset latency, and sleep duration in the PA intervention group [51] (Table 5, at the end of the text).

### Subjects with other health conditions

Two RCTs investigated PA in sleep in subjects with other health conditions, as shown in Table 6, at the end of the text. Cho et al. found that intradialytic exercise, including aerobic exercise, resistance exercise, and combination exercise was clinically beneficial in improving sleep quality via decreasing sleep fragmentation in patients receiving maintenance hemodialysis [64]. Freburger et al. found that a low-to-moderate-intensity PA program in older adults with arthritis resulted in fewer days waking up tired and fewer days waking up at night than in controls [63] (Table 6, at the end of the text).

### Three Major Themes in The Effect of PA on Sleep

#### Theme 1: Environment control for sleep may be as or more important than PA intervention

Only two of the twenty-one studies concluded that PA did not help with sleep. Alessi et al. found that increasing daytime PA alone did not improve sleep, owing to a poor sleep environment [60] but after controlling the nighttime sleep environment, they proved that daytime PA resulted in improved sleep [51]. DuBose and K. Hadi also mentioned that noise and nursing care activities are modifiable causes of some sleep disruptions in hospitals, and when reduced, can lead to more sleep [69]. In another study, Rayward et al. found an adjunctive PA intervention did not improve sleep quality [55]. The APP to aid goal setting and self-monitoring PA and evaluating whether the target has been achieved increased the PA of the subjects. However, there was no difference in sleep quality compared to the group with only sleep hygiene. Such results are likely to be distorted by sleep hygiene behavioral education that improves the sleep environment, and even has the same effect on improving sleep as PA. General sleep hygiene recommendations advise individuals to maintain a cool, dark, and quiet sleeping environment [70]. Cognitive Behavioral Therapy for Insomnia has been demonstrated superior to aerobic exercise in that it resulted in significantly different scores on the Insomnia Severity Index [71, 72]. Although only two studies in our review have shown that sleep environmental control may distort the effects of PA on sleep, this may imply that an environment that provides adequate conditions for sleep prior to PA intervention may be more important for people with sleep disturbance. More research is needed to confirm the above ideas.

#### Theme 2: All PA is efficient for improving sleep in all populations

Across the diverse range of PA interventions, including those given only by APP, web, or actigraphy, PA positively affected sleep satisfaction (sleep quality), efficiency (sleep onset latency, or sleep efficiency), and duration (total sleep time) in almost all populations we reviewed. In our review females dominated, at over 78%. However, the effect sizes of PA on sleep remained medium to large in studies in which females were less than 60% [51, 58, 64]. Therefore, any PA appears to be effective for sleep in both genders and in different populations (Table 7).

#### Theme 3: Self-tolerated PA is safe for improving sleep in working age adults, the elderly, illness and perinatal populations

All of the trials in our review demonstrated that the subjects had high compliance (by QACIS, Additional file 1, & 3), with almost no side effects due to PA, and only one in 3677 participants experienced muscle injury (Additional file 3) [62]. Thus, self-tolerated PA appears to be safe for the human body and even for fragile populations. This conclusion is consistent with a recent consensus, which mentioned that the benefits of physical activity outweigh the risks for people living with long-term conditions [73].

## Discussion

PA showed clear benefits in all populations although sleep outcomes were less consistent in healthy working age adults. The amount and type of PA, the age and gender of the subjects, the presence or absence of disease, and the sleep environment are all variables that affect the impact of PA on sleep.

### Strategies of PA for sleep disturbance

PA over all had positive effects on sleep in all populations, regardless of co-morbid conditions, including PA of varying amounts and types, even just housework (small effect size on sleep onset latency for healthy older women) [45], walking, wheeling or sit-to-stand repetitions (large effect size on sleep efficiency for older adults) [57] . Nguyen et al.’s wearable-based PA intervention increased moderate or vigorous PA and decreased sitting time [68] with a medium effect size on sleep efficiency (sleep efficiency, wake after sleep onset, and PSQI) in breast cancer patients [50]. Web pages, videos and apps that encourage PA were mainly aimed at healthy working age adults, and only had a slight impact on sleep, with effect sizes ranging from none to medium [44, 52–55] (Additional file 3). All the above studies without rigorous surveillance were conducted during the covid 19 pandemic era from 2019 to 2021. Other intervention programs, including behavioral feedback, or goal setting or health coaching programs, provided behavioral support to improve adherence through phone contact, email connect, or group and individual interactions. These interventions, primarily targeted at subjects with co-morbid conditions and primary insomnia, had positive effects on sleep, with effect sizes ranging from small to large [49, 60, 62, 63] (Additional file 3). The studies incorporating strict monitoring for PA dose had a pronounced effect on sleep, with effect sizes ranging from medium to large [47, 48, 51, 61, 64] (Additional file 3). The currently available data also indicated a positive effect of exercise and PA on sleep quality. Both relatively high-intensity and low-intensity exercise produced significant benefits associated with sleep quality [74]. Consistent with this, De Nys et al.’s systematic review and meta-analysis highlighted that increased PA may improve sleep quality in adults with long-term conditions or current poor (mental) health states [75]. Therefore, a more structured, rigorous, and high-volume PA intervention might be expected to achieve larger effect sizes for sleep, mainly on sleep satisfaction (sleep quality and PSQI), and efficiency (PSQI, sleep efficiency, and sleep onset latency). However, PA at any light intensity, such as housework, walking, sit-to-stand repetitions, and even encouraging PA through web pages, videos and app had a positive impact on sleep, especially in the era of the covid 19 pandemic.

### The effect of acute PA on sleep

The effects of PA on sleep can be divided into two types: acute and chronic. The acute one is the impact on sleep after PA on the same day, and the chronic one is the impact on overall sleep after a period of regular PA [17]. According to the literature we reviewed, only one article investigated the effect of acute exercise on sleep; Seol et al. found that low-intensity exercise and housework in the evening induced a positive effect on sleep efficiency (objective sleep latency) [45]. Further, Bission et al. proved that on days with more steps, sleep satisfaction (sleep quality) was better and sleep duration was longer [52]. Consistent with this, Chen et al. found that a single session of light-intensity walking had a positive effect on sleep latency and sleep efficiency in older women with mild sleep impairment [76]. However, it is believed that the effect of regular long-term PA will be more significant. Studies on the acute effects of a single bout of light‐intensity PA on sleep are rare, and more relevant studies should be conducted to confirm this conclusion.

### Effect sizes of PA on sleep in various populations

After summarizing and calculating the effect sizes of various measures across different populations, we found that the largest effect size was achieved in the perinatal population (Table 7; calculations available in the Additional file 3) where PA had a positive effect on all presented outcomes. Both trials in perinatal women demonstrated a large effect on sleep satisfaction (sleep quality and PSQI) [46, 47]. Consistent with this, Choong et al.’ meta-analysis had also concluded that exercise interventions produced significant effects on improving sleep with medium to very large effect sizes among perinatal women [77]. In subjects with cancer, PA had diverse effects on sleep, most of which were of medium to large effect sizes [48–50, 60] (Table 7). Yang et al. conducted a meta-analysis and concluded that PA intervention could improve sleep quality in breast cancer survivors; however, there were no significant differences in PSQI, sleep duration, sleep latency, habitual sleep efficiency, and daytime dysfunction between intervention and control groups, not compatible with our review [78]. Yang et al. include only six articles, 2 of which PA had no appreciable effect on PSQI, which may be related to the sealing effect, as the subjects' sleep disturbance was not severe enough and the baseline PSQI total score was around 5 to 6. In the other four articles, PA had a clear effect on PSQI, consistent with our results. Last, in the healthy working age population, three trials revealed a small to medium effect, two on sleep quality and PSQI, and one on sleep efficiency [52–54].

Overall, PA had a positive effect on sleep in all populations in our review, with greater effects in perinatal women and older adults. However, in healthy working age populations, the effect sizes were smaller.

### Measurements of sleep

As mentioned above, Rayward et al. found that adjunctive PA did not improve sleep quality [55]. It is possible that such results may have been affected by the use of subjective questionnaires. Another possibility is that behavioral education on sleep hygiene improved the sleep environment with an equivalent effect as PA on sleep. If we wish to differentiate between these possibilities, objective sleep measurements such as actigraphy or polysomnography should be considered. Aili et al. found a low correlation between actigraphy sleep parameters and subjective sleep quality, suggesting that the two methods of measurement capture different dimensions of sleep [79]. Likewise, Hughes et al. found no agreement between subjective and objective sleep assessment; compared with objective measures, half of participants reported worse sleep efficiency on questionnaires [80]. Fabbri et al. found that the self-report questionnaires assessing sleep quality from different perspectives have high internal consistency and test-retest reliability [81]. Therefore, clinicians should consider utilizing both objective and subjective sleep measures to identify individuals who may benefit from PA as a treatment for sleep disturbance.

### Effect of PA on various sleep measurements

In our review, PA trials were more effective in improving sleep efficiency. In practice this may be partly explained by the reductions in sleep latency, wake after sleep onset, and sleep fragmentation but we could not find the relationship in our review. Yang et al. mentioned that the effects of exercise might depend on the type of insomnia (sleep initiation or maintenance) [82]. Improvement in sleep latency might be more beneficial for sleep initiation, and reduction in wake after sleep onset and sleep fragmentation might be more beneficial for sleep maintenance. There is still no relevant literature about the effect of PA on sleep initiation or maintenance; further studies should focus on subjects with sleep initiation impairment, or sleep maintenance impairment, respectively.

### Strengths and limitations

In this scoping review, a rigorous, comprehensive search strategy across four databases was used to identify relevant articles conforming to the study criteria. Furthermore, a systematic, in-depth data extraction process was performed in duplicate to ensure reliability. Despite these strengths, this review has several limitations. The first limitation is the selection bias. Studies may not have the word “physical activity” in the title and may include “walking” or “daily activity.” Second, we did not include gray literature, which increased the scientific consistency but may have reduced the breadth and depth of the articles included. Third, as with approximately 60% of all scoping reviews, we did not have the opportunity to consult with stakeholders, and may thus have omitted some research gaps that need to be filled [83]. Additionally, one effect size could not be obtained from the available data for the calculation in our review (Alessi et al. (1999) [57]) and we therefore included it from a meta-analysis in the literature [17]. We cannot compare the effect size from a meta-analysis in the literature with our other effect sizes. Lastly, the results of this study may have been influenced by the search terms that were used, the number of databases searched, and the selection of databases used in the search. As a result, the findings of this review may be influenced by publication bias.

## Conclusions

This scoping review provides a map of the literature on the effect of PA on sleep in various populations. PA of various types is an effective and safe non-pharmacological intervention for sleep disturbance in healthy and co-morbid populations. Even PA at low-intensity, such as doing housework, walking, sit-to-stand repetitions, has a positive impact on sleep, as do web pages, videos and apps including self-goal setting. In addition, this scoping review identified that further work is needed to assess which of sleep environmental control and PA intervention is more important for sleep disturbance, and to identify the contribution of PA to impaired sleep initiation or sleep maintenance, respectively, as areas for potential therapeutic research and future exploration.

### Availability of supporting data

The following supporting information can be downloaded at https://www………: Additional file 1: Quality Assessment of Controlled Intervention Studies (QACIS) of the included articles. Additional file 2: Quality Assessment of Controlled Intervention Studies (QACIS) Criteria. Additional file 3: Original or calculation effect size and frequency, intensity, time, type, and duration (FITT-D) of physical activity of the included articles.

## Supplementary Information

**Additional file 1: Table S1.** Quality Assessment of Controlled Intervention Studies (QACIS) of the included articles.

**Additional file 2: Table S2.** Quality Assessment of Controlled Intervention Studies (QACIS) Criteria.

**Additional file 3.** Summary of effect sizes of PA intervention and FITT-D in the included articles.

## Data Availability

The datasets used and analyzed during this review are available from the articles.

## Abbreviations

PA: Physical activity
RCT: Randomized clinical trials
PRISMA: Preferred Reporting Items for Systematic Reviews and Meta-Analysis
QACIS: Quality Assessment of Controlled Intervention Studies
N/A: Not applicable
NHLBI: National Heart, Lung and Blood Institute
SATED: Satisfaction, Alertness (during waking hours), Timing, Efficiency, and Duration
PSQI: Pittsburgh Sleep Quality Index
WASO: Wake after sleep onset
NWAKE: Number of awakenings
FITT-D: Frequency, intensity, time, type, and duration
