# Supplementary material for: The effect of physical activity on sleep disturbance in various populations: a scoping review of randomized clinical trials
Source: Int J Behav Nutr Phys Act. 2023 Apr 17;20:44. doi: 10.1186/s12966-023-01449-7 (PMC10107572; doi:10.1186/s12966-023-01449-7)
Supplement: Supplementary file 1 — Additional file 1: Table S1. Quality Assessment of Controlled Intervention Studies (QACIS) of the included articles. [file 12966_2023_1449_MOESM1_ESM.docx]

| Authors | 1 | 2 | 3 | 4 | 5 | 6 | 7 | 8 | 9 | 10 | 11 | 12 | 13 | 14 | total | % |
| --- | --- | --- | --- | --- | --- | --- | --- | --- | --- | --- | --- | --- | --- | --- | --- | --- |
| Bisson et al. (2019) | 1 | 1 | 1 | 0 | 0 | 0 | 1 | 1 | 1 | 1 | 1 | 1 | 1 | 1 | 11 | 78.57% |
| Murawski et al. (2019) | 1 | 1 | 1 | 0 | 0 | 1 | 1 | 1 | 1 | 1 | 1 | 1 | 0 | 0 | 10 | 71.43% |
| McDonough et al. (2021) | 1 | 1 | 1 | 0 | 1 | 1 | 1 | 1 | 1 | 1 | 1 | 1 | 1 | 1 | 13 | 92.86% |
| Rayward et al. (2020) | 1 | 1 | 1 | 0 | 1 | 1 | 1 | 1 | 1 | 1 | 1 | 1 | 0 | 1 | 12 | 85.71% |
| Chee et al. (2019) | 1 | 1 | 1 | 0 | 0 | 1 | 1 | 1 | 1 | 1 | 1 | 0 | 1 | 1 | 11 | 78.57% |
| Alessi et al. (1995) | 1 | 0 | 1 | 0 | 1 | 1 | 1 | 1 | 1 | 1 | 1 | 1 | 1 | 1 | 12 | 85.71% |
| Alessi et al. (1999) | 1 | 0 | 0 | 0 | 0 | 1 | 1 | 1 | 1 | 1 | 1 | 0 | 1 | 1 | 9 | 64.29% |
| Seol et al. (2021) | 1 | 1 | 1 | 0 | 0 | 1 | 1 | 1 | 1 | 1 | 1 | 1 | 1 | 1 | 12 | 85.71% |
| Bademlim et al. (2019) | 1 | 1 | 1 | 0 | 0 | 1 | 1 | 1 | 1 | 1 | 1 | 1 | 1 | 1 | 12 | 85.71% |
| Vazfragoso et al. (2015) | 1 | 1 | 1 | 0 | 1 | 1 | 1 | 1 | 1 | 1 | 1 | 1 | 1 | 1 | 13 | 92.86% |
| Rodriguez-blanque et al. (2018) | 1 | 1 | 1 | 0 | 0 | 1 | 1 | 1 | 1 | 1 | 1 | 1 | 1 | 0 | 11 | 78.57% |
| Hawkins et al. (2019) | 1 | 0 | 0 | 0 | 0 | 1 | 1 | 1 | 1 | 1 | 1 | 0 | 1 | 0 | 8 | 57.14% |
| Freburger et al. (2010) | 1 | 1 | 1 | 0 | 0 | 1 | 1 | 1 | 1 | 1 | 1 | 0 | 0 | 1 | 10 | 71.43% |
| Cho et al. (2018) | 1 | 1 | 1 | 0 | 0 | 1 | 0 | 0 | 0 | 1 | 1 | 0 | 0 | 0 | 6 | 42.86% |
| Nguyen et al. (2021) | 1 | 1 | 1 | 0 | 0 | 1 | 1 | 1 | 1 | 1 | 1 | 1 | 0 | 0 | 10 | 71.43% |
| Rastogi et al. (2020) | 1 | 1 | 1 | 0 | 0 | 1 | 1 | 1 | 1 | 1 | 1 | 1 | 1 | 0 | 11 | 78.57% |
| Rogers et al. (2017) | 1 | 1 | 1 | 0 | 1 | 1 | 1 | 1 | 1 | 1 | 1 | 1 | 1 | 0 | 12 | 85.71% |
| Roveda et al. (2017) | 1 | 0 | 0 | 0 | 0 | 1 | 1 | 1 | 1 | 1 | 1 | 1 | 1 | 1 | 10 | 71.43% |
| Hartescu et al. (2015) | 1 | 1 | 1 | 0 | 0 | 1 | 1 | 1 | 1 | 1 | 1 | 1 | 1 | 1 | 12 | 85.71% |
| Wang et al. (2015) | 1 | 1 | 1 | 0 | 1 | 1 | 1 | 1 | 1 | 1 | 1 | 0 | 1 | 1 | 12 | 85.71% |
| Tse et al. (2019) | 1 | 1 | 1 | 0 | 1 | 1 | 1 | 1 | 1 | 1 | 1 | 1 | 1 | 0 | 12 | 85.71% |

Table S1 Quality Assessment of Controlled Intervention Studies (QACIS) of the included articles.

|  | Evaluation item |
| --- | --- |
| 1 | Was the study described as randomized, a randomized trial, a randomized clinical trial, or an RCT? |
| 2 | Was the method of randomization adequate (i.e., use of randomly generated assignment)? |
| 3 | Was the treatment allocation concealed (so that assignments could not be predicted)? |
| 4 | Were study participants and providers blinded to treatment group assignment? |
| 5 | Were the people assessing the outcomes blinded to the participants' group assignments? |
| 6 | Were the groups similar at baseline on important characteristics that could affect outcomes (e.g., demographics, risk factors, co-morbid conditions)? |
| 7 | Was the overall drop-out rate from the study at endpoint 20% or lower of the number allocated to treatment? |
| 8 | Was the differential drop-out rate (between treatment groups) at endpoint 15 percentage points or lower? |
| 9 | Was there high adherence to the intervention protocols for each treatment group? |
| 10 | Were other interventions avoided or similar in the groups (e.g., similar background treatments)? |
| 11 | Were outcomes assessed using valid and reliable measures, implemented consistently across all study participants? |
| 12 | Did the authors report that the sample size was sufficiently large to be able to detect a difference in the main outcome between groups with at least 80% power? |
| 13 | Were outcomes reported or subgroups analyzed prespecified (i.e., identified before analyses were conducted)? |
| 14 | Were all randomized participants analyzed in the group to which they were originally assigned, i.e., did they use an intention-to-treat analysis? |

Table S2 Quality Assessment of Controlled Intervention Studies (QACIS) Criteria
